# *Deep listening*: towards an imaginative reframing of health and well-being practices in international development

**DOI:** 10.1080/17533015.2013.827227

**Published:** 2013-08-13

**Authors:** Mercédès Pavlicevic, Angela Impey

**Affiliations:** a Nordoff Robbins Music Therapy, London, UK; b School of Oriental and African Studies, University of London, London, UK

**Keywords:** international development, health and well-being practices, Community Music Therapy, applied ethnomusicology, deep listening

## Abstract

This paper challenges the “intervention-as-solution” approach to health and well-being as commonly practised in the international development sector, and draws on the disciplinary intersections between Community Music Therapy and ethnomusicology in seeking a more negotiated and situationally apposite framework for health engagement. Drawing inspiration from music-based health applications in conflict or post-conflict environments in particular, and focusing on case studies from Lebanon and South Sudan respectively, the paper argues for a re-imagined international development health and well-being framework based on the concept of *deep listening.* Defined by composer Pauline Oliveros as listening which “digs below the surface of what is heard … unlocking layer after layer of imagination, meaning, and memory down to the cellular level of human experience” (Oliveros, 2005), the paper explores the methodological applications of such a dialogic, discursive approach with reference to a range of related listening stances – cultural, social and therapeutic. In so doing, it explores opportunities for multi-levelled and culturally inclusive health and well-being practices relevant to different localities in the world and aimed at the re-integration of self, place and community.

## Introduction: Whose Health? Whose Art? Whose Development?

International development was established as a professional sector in the USA shortly after the Second World War as an attempt to redress growing poverty in the colonised or former colonial dependencies in the world (Escobar, 2001, Allen & Thomas, 2000). What began as an economic initiative, driven by Eurocentric notions of growth, progress and modernisation, has since been transformed into a vast indfustry that operates across a range of infrastructural, policy and social concerns.^1^ Although the prescriptive proclivity of the development agenda has provoked much critical re-framing over the years, directed largely through a language of partnerships, participation and citizen rights, there nevertheless remains vigorous debate around the ongoing exclusion of local social and cultural practices from the purview of international development programme agendas, and equally, about the general disregard demonstrated by international agencies towards culturally apposite modes of programme implementation and communication.

This paper will focus on the international development health and well-being agenda specifically, drawing particular attention to its application in conflict or post-conflict environments. It extends the notion that international development health policies remain reluctant to take on board cultural understandings of health and healing, which are often embedded in a broad ecology of social, spiritual and physical maladies and managed in a context of ritualised expressive action. As a result, programmes established by international development health agencies create asymmetries of health “currencies,” importing practices from the global North and relegating local cultural understandings, practices and practitioners to the margins.

In an attempt to counter this disjuncture, the paper draws on the conceptual and methodological paradigm of *deep listening* as proposed by composer Pauline Oliveros, extending its conceptual foundation as a form of listening that “digs below the surface of what is heard … unlocking layer after layer of imagination, meaning, and memory down to the cellular level of human experience” (Oliveros, 2005). Fundamental to *deep listening* are Oliveros’ considerations of the place of individual sounds within their acoustical environment, linked to an awareness of the constantly shifting relations that occur between the individual and the collective. From this, we infer a correspondence with a variety of “listening stances” in proposing a re-imagined, more culturally apposite framework for music/arts-based health interventions. Such a framework would privilege a reflexive approach based on critical responsiveness to the intimacies of situated social interactions and to the cultural meanings assigned to them in particular times and places.

The first *deep listening* stance we propose applies to “cultural listening,” a concept that is central to the practice of ethnomusicology, which uses as its point of entry critical enquiry into the cultural practices and meaning systems of a group, and focuses in particular on the ways in which music is situated and experienced within this frame. To this we add the concept of “social listening,” supported by music sociologist Tia DeNora’s (2000, 2011) work on everyday life *musicking* and on the musicality of social exchanges, patterns and spaces. “Social listening,” we suggest, responds to shared performance and improvisation as embodied social patterns and configured social spaces, and conversely listens to social and acoustic patterns and engagements as music. Finally, “therapeutic listening” draws from the listening between therapists and clients, to reflect on, and respond to, the way individuals and groups listen to different aspects of themselves, to the relationships between them and listen to being listened to (Verney & Ansdell, 2010).

To illustrate these stances, we explore the concept of *deep listening* in relation to two contrasting case studies, each focusing on a country that would fall under the UK Department of International Development’s designation of a “fragile and conflict affected state.” Work in such environments lends urgency to health interventions, bringing into particularly sharp focus the need for critical attentiveness to local responses and coping mechanisms. The first case study reflects on an arts-based therapy workshop (following the 2006 Israeli–Hezbollah war) held with Lebanese health workers whose lives are demarcated by protracted low-level conflict and sudden high-intensity war. In this context, we invoke *deep listening* as a retrospective analysis – describing negotiated arts interventions that thread together all three stances, navigating between the individual, collective, cultural and political. The second focuses on the recent aftermath of a protracted high-level conflict in South Sudan, offering an ethnomusicological analysis of Dinka song and performance, and advocating their use as the basis for potential arts-based interventions for post-traumatic stress.

## Disciplinary Perspectives

The disciplinary convergence of Community Music Therapy and ethnomusicology emerged from our shared teaching on the MA Music in Development Programme at the School of Oriental and African Studies (SOAS), University of London, with a lively group of students, many of whom are musicians with substantial experience in community-based cultural practices and some with international development agencies abroad. This teaching sharpened our understanding of the differences, similarities and overlaps between what might be loosely termed as “applied” or “development” ethnomusicology and music therapy, and whose theoretical frameworks and professional practices are rooted in the “global North.” While tackling issues of participatory arts and health practices from apparently asymmetrical discursive perspectives, we came to recognise that while some degree of ethnocentrism and cultural imposition in arts and health practices (and scholarship) is unavoidable, reflexive, culturally tempered and responsive listening is essential to participatory practice.

The “modern” discipline of music therapy emerged as a western professional practice towards the end of the Second World War, with the exploration of new treatments for wounded and distressed soldiers. Detailed empirical observation and analysis of negotiated spontaneous shared musical improvisation between musician-therapists and people “in need” (whether identified by western medical manuals such as DSM-IV, or as a result of social transgressions) have spawned a robust theoretical basis for Improvisational Music Therapy (Ansdell, 1995; Bruscia, 1987; Nordoff & Robbins, 2007; Pavlicevic, 1997, 1999; Pavlicevic & Ansdell, 2009; Ruud, 1998; Wigram, 2004).

Based on an understanding that shared musical participation is itself constitutive of individual and collective bio-psycho-social health (MacDonald, Kreutz, & Mitchell, 2012; Malloch & Trevarthen, 2009; Pavlicevic, 2003), Community Music Therapy emerged in the new millennium as an international countercultural professional discourse, on the basis of a growing range of music therapy practices in non-medicalised settings in various parts of the world. Community Music Therapy challenges the explicitly medical/therapeutic frames that seems to have constrained the natural social effervescence of shared musical practices, and signalled a (somewhat belated) turn from the “medical – therapeutic” – to the “social” nature of music therapy as a health practice (Ansdell & DeNora, 2012; Pavlicevic & Ansdell, 2004; Stige & Aaro, 2012; Stige, Ansdell, Elefant, & Pavlicevic, 2010).

Until recently, ethnomusicological studies on healing focused predominantly on the role of music and dance in healing processes (e.g. trance), drawing specifically on a culturally embedded understanding of health and healing as advocated by cultural anthropology (see Becker, 2004; Berliner, 1978; Friedson, 1996; Gouk, 2000; Rouget, 1985). Over the past five years, however, medical ethnomusicology has begun to emerge as a distinct research and applied practice, mobilised in large parts by the AIDS pandemic, where music has been actively incorporated into a spectrum of communication initiatives to support healthcare programmes in different localities in the world. According to Koen:

medical ethnomusicology … focuses specifically on music and sound phenomena and the roles they play in any context of healing. Such roles can be physical, mental, emotional, spiritual and social in nature. As a research track within ethnomusicology, medical ethnomusicology encourages integrative research, in which collaboration between experts from a broad diversity of fields, including music, medicine, health science, cultural and comparative studies is at times essential to explore holistically issues of music and healing. (2008, p. 28)

Two narrative case studies, drawn from our direct experiences of music-based health applications in conflict or post-conflict environments – in Lebanon and South Sudan – offer a prism for elaborating on the stance of *deep listening*, which draws specifically on the cultural wisdoms and potencies of local health and arts currencies.

## Case Study 1

### The Firemaker Lebanon Project: Negotiating Impositions and Interventions

This project (narrated by the first author) fits within the international development model that draws on “experts” from the affluent global North entering a local arena and imparting expertise – through, in this instance, an arts-based participatory workshop facilitated by three of us (the training team) with health/care workers in Lebanon. *Deep listening* is signalled in this account through the constant negotiation by the entire workshop group^2^ on structure, delivery and workshop values, and the workshop participants themselves considering and deciding how the workshop experiences would (and would not) translate into their everyday lives.

In the northern spring of 2007, a four-day residential experiential training in the arts-as-healing practices for healthcare professionals took place in the *Chouf* Mountains of Lebanon. Organised by a Beirut-based NGO (*Aid Lebanon)*, the training was negotiated through a local representative and provided by three of us: a team of western-trained arts therapy practitioners based in South Africa and London (Music, Drama and Drama-Play). All of us trained in the UK and had considerable international experience of doing similar work in formal and informal healthcare and urban/rural community settings in the “developing” world both in sub-Saharan Africa and South America. In addition, I am familiar with Lebanon, having spent part of my childhood there and visiting a relative regularly. Preparations for *Firemaker* were as consultative as practically possible, given the intercontinental distances. As a training team, we planned the four-day training structure, mindful of the possibilities – and indeed probabilities – of the multiple and refracted “otherness,” both between the participants and also between the participants and ourselves. In the days before the training, we spent time together in Beirut eliciting from locals norms concerning language, translations, religious groupings, gender issues and regional variations in sensitivities and experiences of conflict (e.g. the South of Lebanon was occupied by Israeli forces until 2000, the South of Beirut was the prime target in the 2006 Hezbollah war with Israel, while the North of the country is more entangled in Syrian affairs).

The first half-day of the workshop was devoted to introductions and expectations, as well as clarifying the limits of the training and an overview of the schedule for our time together. What became clear in these first hours was the participants’ range of professional experiences and world views. All participants were locally trained in “western” health and education practices (physiotherapist, social workers, psychologist, teachers, psychiatrist) and some had done additional studies in Western Europe (UK and France) and North America. While many had strong regional and religious identities, others (who returned to Lebanon as young professionals relatively recently, their families having settled elsewhere during the civil war) described themselves as having multiple national identities: American/Canadian-Lebanese, French-Lebanese, etc. This multiplicity of identities reflects the complexities of a nation where religious and regional clan identities often override that of a coherent national Lebanese identity (Larkin, 2011; Levin, Henry, Pratto, & Sidanius, 2003; Mohammad-Avvali, 2005).

Arabic and English were the negotiated languages for the workshop, with agreements reached regarding the content and limits of translations for us as non-Arabic speakers. We agreed that not everything would be translated. In addition, the entire group negotiated the norms for participating: we agreed on punctuality, no sub-conversations and – most difficult of all – mobile phones on silent. Given the volatile political situation of the region,^3^ we settled on participants checking their phones at regular intervals and leaving the room to return what seemed to be emergency calls.

From the start, the participants made it clear that they wanted regular and frequent discussion time during the four days to translate workshop experiences into their daily work situations, rather than doing this at the end of each day as we had planned. During the negotiation of workshop expectations, the participants’ musical/artistic energy emerged, together with their lack of practical experience in the use of the arts as health intervention. As a group we established that the training team would depend on the participants to constantly review what might and might not be appropriate and/or useful in the workshop, for the healthcare professionals’ own practices, given the local norms and contexts.

Following the negotiations of shared norms, a first “exercise” sought to elicit what aspects of life in Lebanon might be most usefully addressed by the arts. The participants emphasised the mental – social collective consequences of living in an uncertain environment – and de-emphasised the physical symptoms of stress. They described Lebanese people generally as having “a well-developed sense of helplessness” – and although we did not experience this as falling within a “victim” frame, there was a palpable sense of frustration, fragility and at times, of depression and suffering within the workshop participants (some of whom had seen their homes demolished and lost relatives in the 2006 war between Hezbollah and Israel).

The participants were alert to what they named the “psychosocial” impact of living in a low-intensity conflict zone, and described how the fragility of notions of “continuity” and the constant threat of interruption to everyday life result in exhaustion, self-defeating thoughts, depression and fearfulness; and on an emphasis on “superficiality” of social displays. When invited to elicit what they considered might be helpful or useful in addressing the kind of ongoing threats of destabilisation, they spoke of resilience and characterised it as the need to “bend but not break,” to have supportive networks and to “re-channel negative energy into positive energy.” (This resonates with Castleden, McKee, Murray, & Leonardi’s [2011] systematic literature review, which describes adaptability and flexibility as key to individuals and communities coping with disturbance and adversity.)

How, then, we asked, might our experiences and knowledge be most useful and relevant during this time we had together? What could the training contribute that the workshop participants did not already know and use? The arts, they responded, might offer “different ways of saying the same thing,” opportunities to be “non-judgemental,” to reflect who they are and to carry many levels of meaning.

An additional issue that emerged in the opening discussions was the risk of the participants’ own burnout and exhaustion, and their need for self-care and nourishment, while also seeking ideas to implement in their work. We agreed as a group to attend to these during the four days.

#### Deep Listening Commentary

As the training team, we witness the participants’ portrayal of their lives from the macro-geopolitics to its social collective and personal consequences. We listen attentively to the language, the terms used, how the participants themselves frame their needs and resources, and their workshop preferences. Our listening stance is multidimensional. This constant awareness and concentration becomes an implicit norm, signalling to the group the equal value of each person’s contribution in the workshop. The first “conversation” seeks to elicit the “buried” systems and resources within the group – that have become forgotten or relinquished as a consequence of ongoing regional instability. Through valuing risks, resources and needs, we signal respect towards people’s experiences, inviting partnership through eliciting their resilience as well as their vulnerabilities. This is *deep listening*, beneath the surface, to what is said and unsaid, overt and covert. The first conversation also signals our collaborative quasi-improvisatory stance: while clear about the expertise that we have to offer from our experiences, the delivery of the workshop can be framed as a shared improvisation, where an emergent structure is collaboratively reviewed and revised. The stance here signals a shared, deftly navigated reflexive journey that takes into account local ecologies of health and well-being, in contrast to the prescribed and imported indicators.

Two musical events are now described in some detail. One concerns introducing the use of songs that are in the public domain, and the other, a brief spontaneous group music improvisation.

#### Event 1

Having agreed that this exercise might well be a useful resource, the participants are clustered into small groups of 5–6, each around a CD player, and each person is invited to present to their group a recorded piece of music that they have especially chosen for this excercise. They are invited to describe – in however much or little detail they wish – the circumstances through which the music has acquired special significance. Animated discussions are punctuated by attentive listening to the selected recordings, with laughter, tears and occasional singing along. Following the listening in small groups, the entire group considers the quality of their listening to one another presenting their music, to the music itself and to their shared comments afterwards.

The choice of music is described as affording each person a representation of “who I am here and now, who I might want to be, who I can hide behind,” within the context of constant fragmentations (or threat thereof) of what it means to be a person, a health/education professional in Lebanon today. As both “listeners” and “speakers,” the participants comment on how they have learnt things about one another that they would not have known otherwise, and how this experience made them feel much closer to one another. The group reflects on how their experiencing of the various songs adds a layer of meaning and association, building memories for future listening (that was X’s song at that workshop). These layers of meaning build on existing national and cultural associations; some chose traditional Lebanese singers (Fay Ruz and Wadi el Saafi), signalling ancestral collective ethnic and regional identity, while others chose younger contemporary Lebanese songs (by May Nasr, Nadine Khoury) with a strong western pop-jazz genre and these contrast to the choice of western pop artists (Rihana).

This was the event that, in the feedback at the end of the workshop, the participants saw themselves applying immediately within their work contexts, where burnout and exhaustion in teams led to people taking themselves and one another for granted.

Three *deep listening* stances are enacted here: as the training team we listen to the general soundscape in the room as musical–social patterning that informs our decisions as to how we might continue or develop this exercise; the participants are guided to listen to one another, to what is unsaid, to what lies within and beyond the surface of the music and to listen to their listening to one another; the entire event is loosely structured (small group listening to each person presenting then playing their chosen music, followed by group reflection and culminating in large group reflection and discussion). Crucially, the participants assign value to this exercise from within their situated experiences – it is they who proffer the usefulness of this exercise for their everyday work, and who identify why and how this musical event might contribute to well-being.

#### Event 2

Immediately following this listening exercise, the participants spontaneously begin making music using the instruments we have made using scrap materials. We listen closely to this impromptu embodied collective “improvisation,” which is a mixture of spontaneous sounds and dance steps, with much cheering, whooping and banging of instruments while dancing into what becomes a circle moving towards and away from the middle. This quickly leads to singing, dancing, clapping and playing traditional Lebanese songs, which we are told are often performed as part of celebrations such as weddings and important feasts. By coincidence, during the drive from the *Chouf* back to Beirut after the workshop, we come across such an occasion, with a wedding party singing and dancing in a – now familiar – genre.

In reflecting after this spontaneous group *musicking*, the animated dancers voice their experiences of “blending in with the group” as “different rhythms being personal,” as “having connections” and as “being part of a group but not in the spotlight.”

#### Commentary

The collective spontaneity seems to be a natural progression, given the emergent workshop ethos of ongoing negotiations and possibilities for spontaneous changes of direction. This felt at the time like an effervescent eruption of participants celebrating their experiences of one another in a new way (through the song-listening experience). The improvised dancing quickly moves towards a more ritualised, shared cultural genre: a traditional folkloric celebration song-dance, when all are “Lebanese,” whatever their regional, religious or national identities. We might speculate here about the health and well-being resonances of such experiences through prisms from the global North: listening to these as the musicalised embodiment of trust, reciprocity, shared participation and an enactment of shared values – generating a sense of belonging and self-worth, of being resourced and of sharing and reconfiguring identities (White, 2009).

This brief event can be seen as a metaphor for a melding of the “new” and the “known,” portraying creative tension and resolution, collectively embodied. However, for this structured spontaneity – and spontaneous emergent structure – to be embodied and enacted, the facilitators remain alert to possibilities for what Sawyer calls the “collaborative emergent” (2000, 2003, 2005). Drawing from jazz and theatre improvisation, he describes groups building on culturally shared resources and repertoire as a spontaneous collective, possible when a group is “in flow.” Collective dancing within a shared cultural frame offers the group opportunities for engaging and connecting with one another, and for embodying and generating shared flow states (Csikszentmihalyi, 1990).

The social imperative for dancing together is not only an expression of collective joy (Ehrenreich, 2007), but it is also understood as having psycho-socio-biological functions. Social bonding and collective identity are enhanced through dancers synchronising their bodies to one another, and sharing a unified experience of space and time (Pavlicevic, 2011). Such shared, situated experiences are immensely powerful in generating group cohesion and building group memory, whatever the differences between group members (Alterhaug, 2004; Ansdell & Pavlicevic, 2005; Clarke, Dibben, & Pitts, 2010; Dissanayake, 2009; Ehrenreich, 2007; MacDonald, Hargreaves, & Miell, 2002; MacDonald et al., 2012; Pavlicevic, 2010; Stern, 2004, 2010). The year for “Alterhaug, 2005” has been changed to “Alterhaug, 2004” to match the entry in the references list. Please confirm that this is correct and provide revisions if needed.

However, crucially, for this apparently spontaneous act to emerge, the entire group needs to remain alert to possibilities for interruptions. Key to the *deep listening* stance is that throughout this spontaneity, the shared and agreed “repertoire” for the workshop remains in focus and open-ended through ongoing opportunities for reflecting on the event and “translating” it into situated contexts. The *Firemaker* narrative brings into focus the delicate balance in threading together local, culturally shaped values with the moment-by-moment here-and-now experiences in the workshop, and inviting participants’ translations of what it means for them to experience social fragility and malaise, of what resources they have and lack and of what the arts might offer them in their everyday work. It is the participants (or in the language of international development, the beneficiaries) who are “in the know” locally.

This approach, we propose, models a re-imagined international development arts intervention: as the training team (on behalf of the “donors”), we invite a co-creation of the workshop frame and repertoire, drawing from the distributed resources and expertise of the entire group (i.e. facilitators and the participants). The resulting social and musical collaboration builds on the group’s similarities (in a country where differences have higher stakes and values), holds diversity of imagination and allows for spontaneity and multiplicity of meanings – much of which is reflected in the assessment at the end of the workshop.

## Case Study 2

### Listening to the Scars of War in South Sudan

South Sudan is the newest country in Africa, having achieved independence in July 2011 following almost half a century of civil war.^4^ Its population comprises some 63 different language groups, the largest of which is the Dinka (*Muonyjieng*), a Western Nilotic people who share close social, linguistic and cultural features with a number of smaller African Nilotic pastoralists, namely the Luo and Nuer. Dinka are scattered across several states in South Sudan, and while the geographical distances between them have resulted in marked linguistic and cultural variation across dialect groups, there nonetheless remains an overriding sense of Dinka identity, which is rooted in common beliefs, values and practices, and reinforced by a shared history of war, trauma and forced migration (Impey, 2013).

Amongst the countless consequences of protracted, high-intensity warfare is the almost complete absence of formal infrastructure and basic social services in South Sudan. This state of affairs is all the more alarming when measured against the extreme social fragmentation and economic deprivation suffered by the majority of its population, a condition that is reflected in some of the lowest human development indicators in the world.^5^ The Government of South Sudan remains heavily reliant on international donors and NGOs to deliver education, healthcare and general infrastructural support. According to the Republic of South Sudan Health Sector Development plan, only 3% of total government expenditure for 2012 went on health (as opposed to 25% on security) and the majority of this allocation was aimed at reducing critically high levels of maternal and infant mortality (Government of the Republic of South Sudan, 2012, p. xxii).^6^

Mental health specialists, Ameresekere and Henderson (2012), caution that while there is an overriding optimism for the future in South Sudan, close attention should be paid to the psychosocial well-being of its citizens. Numerous studies undertaken with South Sudanese, both resident and in the diaspora (the majority of whom are Dinka), relate widespread exposure to violence, displacement and political and social insecurity to post-traumatic stress disorder (PTSD), made evident in high incidences of depression, substance abuse and suicide (Karunakara et al., 2004; Roberts, Damundu, Lomoro, & Sondorp, 2009). Similarly, in her research with South Sudanese refugees in Australia, Tempany (2009) noted that silence, stoicism and suppression are common mechanisms for coping with emotional distress. While sharing experiences with religious or friendship groups may offer some psychosocial release, research has yet to be undertaken to determine whether standard treatments used by western-trained mental health practitioners have been beneficial to members of this population (Tempany, 2009, p. 300). Extending the view proposed by Minas and Silove that “cultural factors can greatly affect the conception, manifestation, diagnosis, subjective experience, and prognosis of mental disorder” (Minas & Silove, 2001, as cited in Tempany, 2009, p. 301), the following two events explore the value of song and dance in Dinka culture in South Sudan, and argues for their consideration as the basis for the development of PTSD treatment.

#### Event 1

I am travelling by road from South Sudan’s new capital city, Juba, to the regional town of Bor, the once administrative centre for the Dinka under the Anglo-Egyptian Condominium (1899–1956) and the birthplace of the second Sudanese civil war (1983–2005).^7^ It is a dry, flat and unremarkable landscape. Heavy vehicles traversing the road during the rainy season have ploughed deep channels into the dark cotton soil that are now compacted into vast sculptured undulations, making for exceptionally arduous driving. Although much has been made about the ongoing insecurity on the border with North Sudan, internal security is still tenuous, often perpetrated by heavily armed and disaffected government soldiers who prey on civilian vehicles at the numerous checkpoints along the way. Yet, while the going is painfully slow and vigilant, I am given a rare opportunity to discuss musical systems with my Dinka research collaborator, who is otherwise in constant demand from church groups, government offices and clan members to participate in an endless succession of meetings in support of the establishment of this fledgling country.

For the past three years I have been conducting academic research on Dinka songs as part of a project entitled “Metre and Melody in Dinka Song and Speech.”^8^ Dinka have a vast taxonomy of song genres that are distinguished from one another by specific musical and performative features and classified according to their social functions. The pervasive use of songs to chronicle individual, group and social life marks them as a kind of “primary symbolic landscape” in Dinka culture, replacing oral discourse in many instances in the public disclosure of certain kinds of information (Deng, 1973). Through song, challenging or distressing experiences are transformed into a subject of “art,” hereby drawing admiration from a wider public and reinforcing respect for the composer and the performers for whom it is intended (Deng, 1973, p. 84; Impey, 2013). As noted by Deng:

Songs everywhere constitute a form of communication which has its place in the social system, but among the Dinka their significance is more clearly marked in that they are based on actual, usually well-known events and are meant to influence people with regard to those events. (1973, p. 84)

Of the large repertoire of songs that we recorded during the project, almost all chronicle – directly or indirectly – events, experiences and sentiments of war. The following song, which is locally classified as *diεt ke t*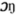 or *diεt ke l*
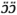
*r* (songs of “spear” or drum) or *diεt ke thiεεεtha* (song of politics), was composed during the second civil war in order to boost the morale of the soldiers in the Southern People’s Liberation Army. It is one of hundreds of similar such songs that were performed by the militia while preparing for battle, and it continues to be sung widely as a memorial to the war effort. As a point of entry into our discussion about the relevance of songs in Dinka society, my research collaborator and I explore this example in greater depth, focusing on two salient features in particular: its value as historical testimony and its place within Dinka song-making culture more generally:

Dr John Garang wanted to govern Sudan

The doctor was in the government of Anyanya One, Sadiq al-Mahdi

It is the land of the Sudan that Garang wanted to govern

The doctor was in the Anyanya One government

We will fight with SAM missiles, Sadiq al-Mahdi.

We will struggle with heavy machine guns

We will fight with kalashnikovs

The guns called BKM and M-46

When our president brought M-46

When our president brought

And he told the officers that they should surrender to the Dinka as the president has arrived.

Garang Mabior is the crowned commander

I am telling you, you Northerners,

If you haven’t already surrendered to the great president of the gun,

*He has now arrived!*^9^

As a chronicle of the civil war, the song provides evidence of the respective protagonists and affirms the political prowess of their beloved leader, John Garang. It also makes evident the widespread and intense militarisation of young South Sudanese, with its attendant (almost mundane) familiarity with heavy weaponry and its allusions to a patriotic fervour that shaped the everyday lives and consciousness of so many for so long. According to my colleague, however, the relevance of such songs goes beyond the mere chronicling of historical experience. “Songs (in general) help us to see and to remember,” he explains; “they are our ‘*miir*’ (divinity); our essence.” “*Miir*” relates equally to indigenous hardwood trees, whose potency is invoked to represent a range of corresponding cultural anchors in Dinka social life such as marriage, worship and language. A song such as this binds people in the immediacy of war, therefore, but more importantly, taps into a deeper acknowledgement of cultural identity and of a collective sense of belonging.

While this song was created for group performance, my colleague explains that most songs are composed to reinforce individual identity and his/her relations with family, clan and wider community. Dinka song making is highly idiosyncratic and is contained within a well-defined ethos of proprietorship. Songs are “owned” by individuals, stored away in a poetic memory bank of relationships, exchanges, places and events, and selectively retrieved for performance at festivals or ceremonies that occur variously in seasonal cattle camps or designated sites beside a village. An individual who lacks the talent to compose good songs will approach a respected song maker to produce a song in exchange for a cow or an agreed sum of money. Occasionally an individual will be considered a talented creator of lyrics only, in which case a second individual, who has an aptitude for good melody making, will be brought into the process. Upon completion, the song will either be taught directly to the “owner,” or, if that person is not a good memoriser, communicated to a group of relatives or age-mates, who will gradually pass it on. Such a division of creative labour extends the role of a song from mere receptacle of information to one that connects individuals to each other and to experiences of particular times and places, hereby implicating individuals in a web of shared aesthetic, social and moral intentionalities.

#### Commentary

Understanding the emotional and social currency of songs in Dinka culture requires a conceptual reframing of the place of song and performance in social life to that generally experienced in the global North. Through the process of deep cultural listening – of literally peeling away the layers of what might otherwise have been considered a mere motivational “war song,” common to many cultures of the world – we come to see how songs act as a culturally sanctioned, multi-sensual, spatial and temporal “site” through which Dinka record, shape and enact their worlds. They are thus the primary medium through which to locate individuals and groups within their social, cultural and physical landscapes. Therefore, to understand song as the emotional, social and cosmological “essence” of Dinka social life and identity affords them a potentially important role in PTSD treatment, providing a culturally legitimate platform for the public disclosure of certain information as well as offering a culturally apposite framework for psychosocial re-integration.

#### Event 2

Given that South Sudan has so recently attained its independence, it is not surprising that the overriding focus of all public rhetoric is that of building a better future. Yet, every Dinka I met during my three-year project seems to be living with memories of profound trauma. One question that plagues me throughout is how individuals and social groups find “normality” beyond the war zone, particularly when war has been the only reality they have ever known.

One day my Dinka colleague pointed to a man in the street and reflected, somewhat nonchalantly:

That man over there is tall like my father.^10^ I had to run away from home when the northern militia attacked our village. At that time I only reached up to my father’s thigh, and when he saw me again, I was taller than him. All those years he didn’t know if I was dead or alive. When we ran, we had to bury our friends along the way. Some of the boys were very small. We saw terrible, terrible things. What are we to do with all those memories that we live with today?

In the ensuing discussion, he tells me about the prevalence of suicide, alcoholism and social withdrawal amongst many of his fellow “lost boys,”^11^ making it evident that South Sudan is awash with individuals suffering from severe, though unattended, PTSD.

Given the chronic underdevelopment of the health services in South Sudan, it is evident that psychosocial care may never be made a priority of the state. Yet I am aware that while everyday conversations about state building are conducted with the same spirit of patriotic zeal as that demonstrated in the song above, when Dinka talk about their songs, their demeanour changes. They sit up straight, smile and immediately engage one in serious discussion. This is not a political conversation; it is one of identity, of individual honour and of his/her place within the wider social and historical locale. It is in his or her songs that a person is truly revealed.

#### Commentary

Given the centrality of songs in Dinka social life, and the value accorded them as a primary source of self-expression and public discourse, it is evident that they could be systematically engaged in the arduous process of post-conflict emotional reparation. Following Olivero’s notion of *deep listening*, two related kinds of “listening” could potentially inform a culturally sensitive framework for the treatment of PTSD amongst South Sudanese as part of international development.

The first focuses on developing a deeper understanding of the role of song in Dinka culture more generally. The vast taxonomy of songs in the Dinka repertory (which comprises distinct categories such as men’s songs, women’s songs, songs of celebration and marriage, insult songs, war and history songs, etc.), as well as the subtle though clearly articulated poetic features that define them provide culturally sanctioned settings for the articulation of common symptoms of PTSD such as fear, sadness, anxiety and disconnection. Songs provide a model of, and for the social reproduction of, individual and social meaning; as they are situated at the intersection between memory, embodiment, representation, materiality and the psyche. They help to reconfigure memories, embodying and enacting a delicate balance between remembering and forgetting. Indeed, within this complex system of song making is a distinct category referred to as “cathartic songs.”

The second kind of listening focuses on a deeper understanding of the inter-subjective nature of song performance and the importance placed on being heard by others as confirmation of one’s identity, experiences and feelings. The use of highly figurative language and conformity to aesthetic practices of melody and bodily gesture are integral to what people remember of other’s songs. It is through these songs that individual identities are articulated and affirmed, but in so doing, they are equally secured within a web of social relations and meanings. In this performative matrix, therefore, is a provision for the expressions of what may otherwise be considered fearfulness, weakness or disconnection and the potential realisation of profound emotional protection by the community of listeners. More than mere renditions of emotions and historical events, therefore, songs tap into a deeply layered and interconnected cultural space, underpinning individuation, belonging and cultural identity in Dinka society.

## Closing Comments

As practitioners and scholars situated in the global North, we are familiar with the emerging discourses clustered under Arts and Health that advocate for the opportunities to experience participatory art-making as constitutive of well-being, whatever our states of “health,” “illness” or “distress.” We are equally familiar with texts from music therapy and from ethnomusicology that portray shared participatory *musicking* as offering opportunities for temporally fluid reorganising of our selves together with others, with corresponding experiences of belonging and social bonding – whether as part of recovering from mental distress, as part of a wedding celebration or as part of a militia approaching battle.

Through our two case studies, we have attempted to elucidate the social potency of a temporally delineated, negotiated participatory arts practice, as well as the possibilities for the incorporation of locally, culturally embedded music practices into health and well-being agendas. In each scenario, the multiple stances of *deep listening* suggest opportunities for shared and negotiated, multi-levelled reframing of people’s experiences, addressing in particular social structural dismantling as transacted by sustained conflict. In each, we have argued that participative *musicking* provides essential social and cultural moorings that help to mobilise and maintain experiences of psychosocial re-integration and (re-)attachment.

Like jazz improvisation, we have argued that the *deep listening* stance holds diversity and seeks to balance the familiar with the unfamiliar, holding tensions and frictions rather than “resolving” and creating “inauthentic” homogeneity, for the sake of imposed health and well-being currencies. In so doing, we have suggested that *deep listening* embraces layers of imagination, meaning and memory that are invoked by sound, while also acknowledging the fluidity, fragility as well as the entrenched symbolic-culture nature of such artefacts.

Finally, on the basis of re-imagining the arts, well-being and development as emergent and dynamic, collaboratively negotiated and improvised, *deep listening* to local currencies of the arts might well help to inform and reframe broader international development agendas and practices. With this assertion, it would seem that locally embedded arts practices belong at the heart – and not the periphery – of international development work as well as its scholarly critiques.

## Footnotes

1 International development encompasses a range of sectors, including foreign aid, governance, health, poverty reduction, gender equality, human rights, economics and environment.

2 Throughout the paper, the “group” includes the facilitators (“the training team”) and the health/care workers (“the participants”).

3 Lebanon’s first large-scale psychiatric epidemiological study, compiled *before* the 2006 war as part of the World Health Organisation World Mental Health Survey Initiative, revealed that 49% of the population sampled had experienced a war-related distressing event of some type (Lee, 2011).

4 Narrated by the second author.

5 Education indicators rate amongst the lowest in the world (e.g. only 27% of the adult population is literate, and less than half of all primary school-aged children attend school (see Government of the Republic of South Sudan, 2011).

6 The infant mortality rate in 2006 was 102 per 1,000 live births, while the maternal mortality rate was 2,054 per 100,000 live births, which is rated the highest in the world (Government of the Republic of South Sudan, 2011, p. viii).

7 The first civil war took place from 1956 to 1973 and was separated from the second civil war by a decade, a period commonly referred to as the “development decade.”

8 The research project was conducted as a collaboration between the University of Edinburgh and SOAS, University of London, with funding from an Arts and Humanities Research Council “Beyond Text” grant. I would like to thank Bob Ladd, Bert Remijsen, Elizabeth Achol Ajuet Deng, Simon Yak Deng Yak and Peter Malek for their invaluable contribution to this research.

9 Ee baai Thudän yen awïc Dïktor Joon Gärä bï kuum (Dr John Garang wanted to govern Sudan) [Juba, South Sudan, July 2011 – Composer: BolDeng recorded solo with *rababa* (lyre)].

10 The Dinka are amongst the tallest people in the world and place great esteem on height.

11 The term “lost boys” was given to the many thousands of young boys aged 7–17 years old who fled on foot to refugee camps in Ethiopia and later northern Kenya to avoid targeted persecution by the northern militia.
